# Supplementary material for: On-field Gross Morphology Evaluation of Dromedary Camel (Camelus dromedarius) Fetal Membranes
Source: Animals (Basel). 2024 May 24;14(11):1553. doi: 10.3390/ani14111553 (PMC11171016; doi:10.3390/ani14111553)
Supplement: Supplementary file 1 [file animals-14-01553-s001.zip › Supplementary file S1. placenta evaluation form.pdf]

PLACENTA EVALUATION FORM

ID DAM................ MOTHER/RECIPIENT............... ID DONOR .... ID BULL ....

DELIVERY EXPULSION TIME .... PLACENTAL EXPULSION TIME ....

DELIVERY spontaneou☐ assis☐d Time from expulsion to evaluation ....

CALF dead ☐ alive ☐ weight .... Kg Gender **M** **F**

Length of the pregnant horn	**LPH**	________
Cranial diameter of the pregnant horn	**CDPH**	________
Middle diameter of the pregnant horn	**MDPH**	________
Caudal diameter of the pregnant horn	**CaDPH**	________
Length of the non-pregnant horn	**LNPH**	________
Cranial diameter of the non-pregnant horn	**CDNPH**	________
Middle diameter of the non-pregnant horn	**MDNPH**	________
Caudal diameter of the non-pregnant horn	**CaDNPH**	________
Length of the umbilical cord	**LUC**	________

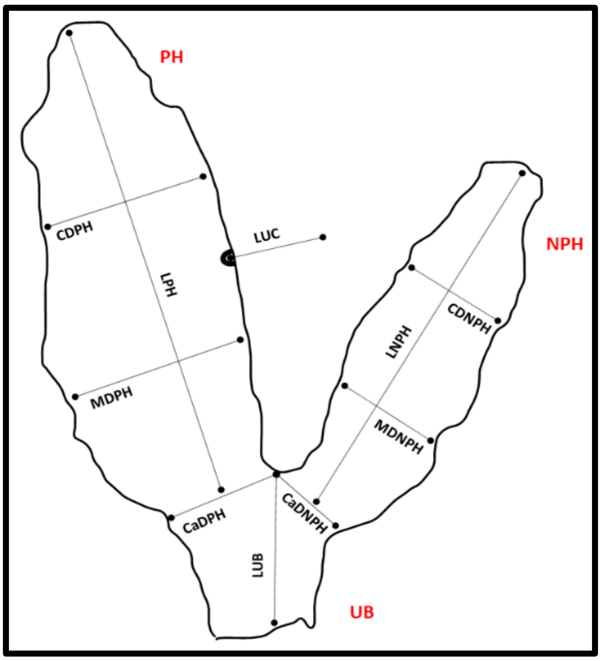

Tara of the bucket ……Kg Total weight ....

Avillous area * Photo ID Avillous area * ....

Necrotic area ● Photo ID Necrotic area ●........................

Histopathology Sample ☐

Histopatology samples ID Histopatology samples Photo ID

Placenta Photo ID

PH Volume ....

NPH volume ....

BODY volume ....

UC volume ....

Total Volume ....

* ● ☐ Draw an asterix, a dot or a square, on the sketch, in the corresponding point of an avillous area, of a necrotic area and in the area where histopathological samples were collected, respectively; write number inside the squares corresponding to the collected samples' identification. In case pictures of specific area are collected, mark the sketch and record the Photo ID

Notes (Overall aspect, storage conditions, any observed abnormalities

....

....

....

....
