# Supplementary material for: On-field Gross Morphology Evaluation of Dromedary Camel (Camelus dromedarius) Fetal Membranes
Source: Animals (Basel). 2024 May 24;14(11):1553. doi: 10.3390/ani14111553 (PMC11171016; doi:10.3390/ani14111553)
Supplement: Supplementary file 1 [file animals-14-01553-s001.zip › Supplementary material.pdf]

**Supplementary material**

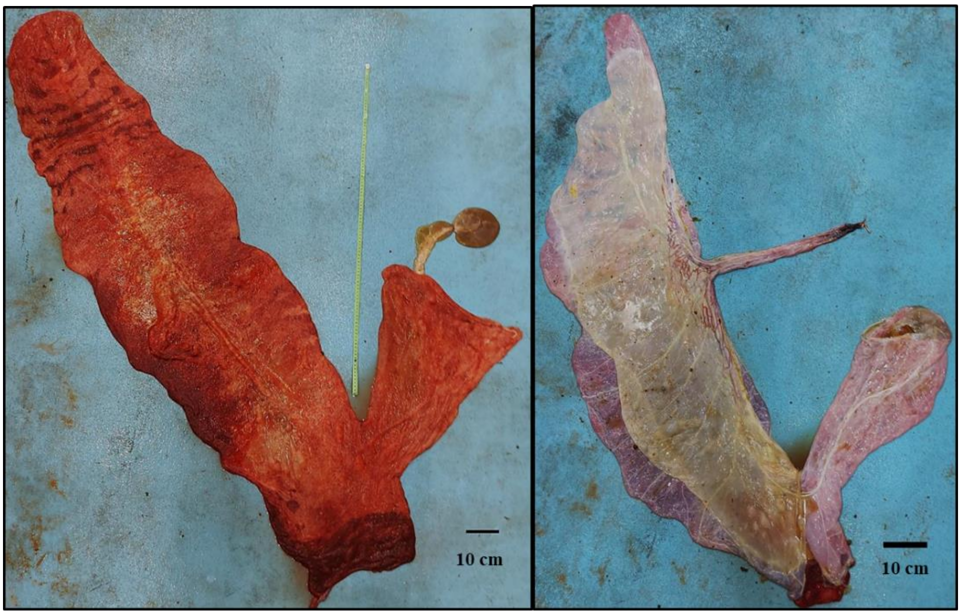


**Figure S1.** Dromedary camel foetal membranes: tearing at the middle of the non-pregnant horn portion. The retained part was expelled 2 hours after the delivery of the calf. Note the less frequent position of the amniotic sac, on the dorsal part the allantoic pregnant horn.

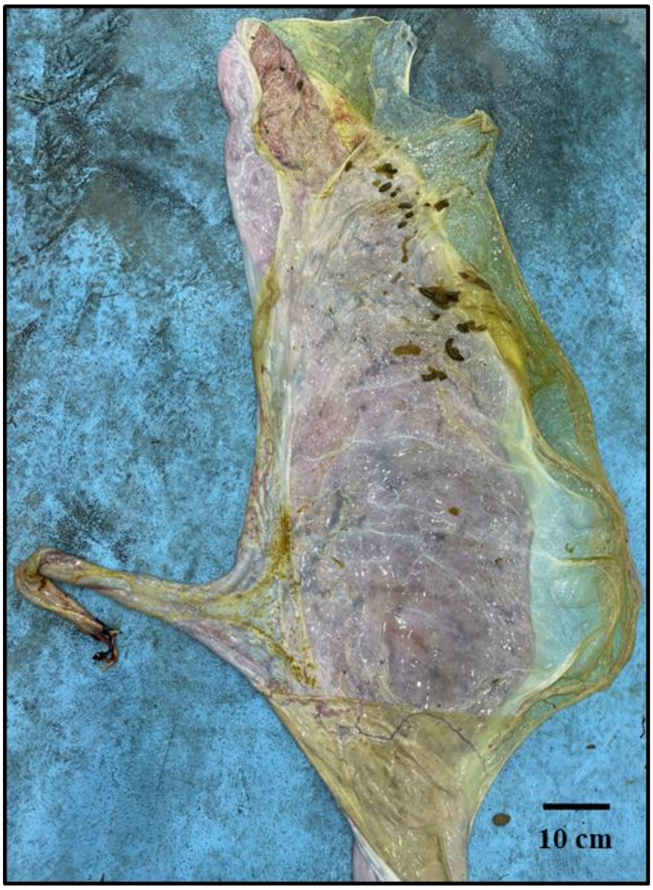


**Figure S2.** Dromedary camel foetal membranes: yellow-green colour of the amniotic sac, due to the presence of meconium, indicating fetal stress during parturition.

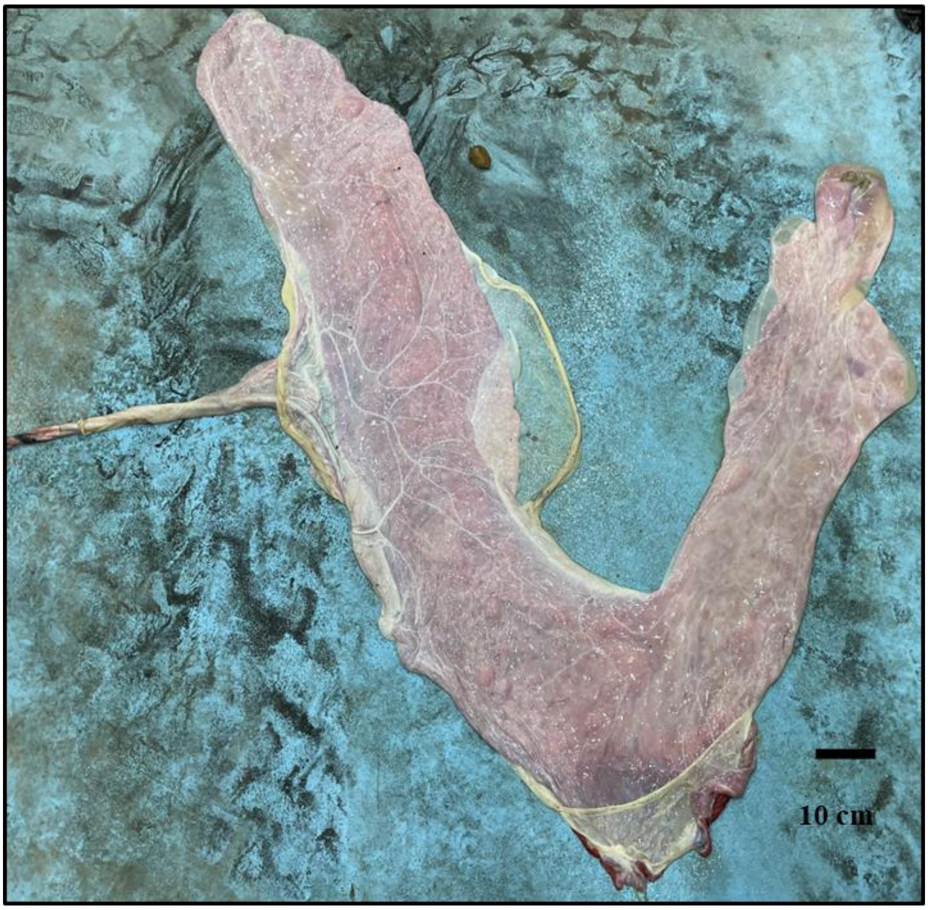


**Figure S3.** Dromedary camel foetal membranes: attachment of the umbilical cord at the lateral side of the pregnant horn allantoic side.

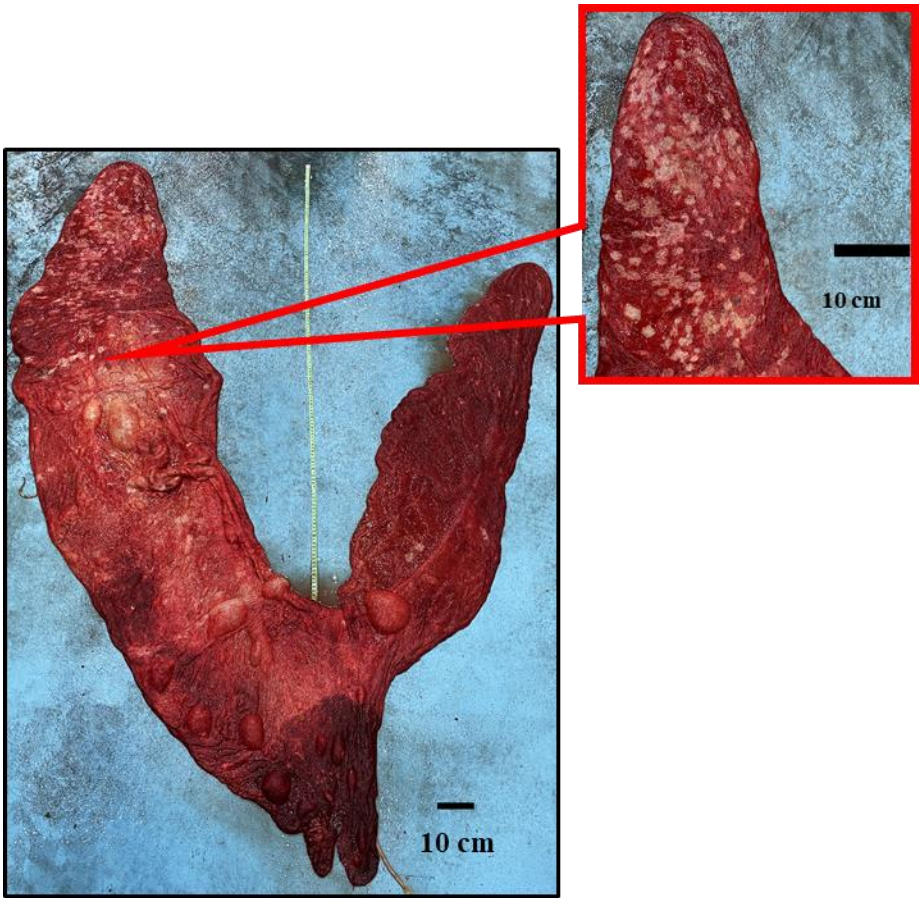


**Figure S4.** Dromedary camel foetal membranes: diffuse multiple focal round-shaped avillous areas of 1-2 cm diameter. The newborn calve was alive and health.
